# Machine Learning for Diagnosis of AD and Prediction of MCI Progression From Brain MRI Using Brain Anatomical Analysis Using Diffeomorphic Deformation

**DOI:** 10.3389/fneur.2020.576029

**Published:** 2021-02-05

**Authors:** Ali Haidar Syaifullah, Akihiko Shiino, Hitoshi Kitahara, Ryuta Ito, Manabu Ishida, Kenji Tanigaki

**Affiliations:** ^1^Molecular Neuroscience Research Center, Shiga University of Medical Science, Shiga, Japan; ^2^Center for the Epidemiological Research in Asia (CERA), Shiga University of Medical Science, Shiga, Japan; ^3^Department of Radiology, Shiga University of Medical Science, Shiga, Japan; ^4^Department of Neurology, Shimane University, Shimane, Japan; ^5^Research Institute, Shiga Medical Center, Shiga, Japan

**Keywords:** artificial inteligence, cognitive impairment, Alzheheimer's disease, machine learning, support vector machine, magnetic resonance imaging, ADNI

## Abstract

**Background:** With the growing momentum for the adoption of machine learning (ML) in medical field, it is likely that reliance on ML for imaging will become routine over the next few years. We have developed a software named BAAD, which uses ML algorithms for the diagnosis of Alzheimer's disease (AD) and prediction of mild cognitive impairment (MCI) progression.

**Methods:** We constructed an algorithm by combining a support vector machine (SVM) to classify and a voxel-based morphometry (VBM) to reduce concerned variables. We grouped progressive MCI and AD as an AD spectrum and trained SVM according to this classification. We randomly selected half from the total 1,314 subjects of AD neuroimaging Initiative (ADNI) from North America for SVM training, and the remaining half were used for validation to fine-tune the model hyperparameters. We created two types of SVMs, one based solely on the brain structure (SVMst), and the other based on both the brain structure and Mini-Mental State Examination score (SVMcog). We compared the model performance with two expert neuroradiologists, and further evaluated it in test datasets involving 519, 592, 69, and 128 subjects from the Australian Imaging, Biomarker & Lifestyle Flagship Study of Aging (AIBL), Japanese ADNI, the Minimal Interval Resonance Imaging in AD (MIDIAD) and the Open Access Series of Imaging Studies (OASIS), respectively.

**Results:** BAAD's SVMs outperformed radiologists for AD diagnosis in a structural magnetic resonance imaging review. The accuracy of the two radiologists was 57.5 and 70.0%, respectively, whereas, that of the SVMst was 90.5%. The diagnostic accuracy of the SVMst and SVMcog in the test datasets ranged from 88.0 to 97.1% and 92.5 to 100%, respectively. The prediction accuracy for MCI progression was 83.0% in SVMst and 85.0% in SVMcog. In the AD spectrum classified by SVMst, 87.1% of the subjects were Aβ positive according to an AV-45 positron emission tomography. Similarly, among MCI patients classified for the AD spectrum, 89.5% of the subjects progressed to AD.

**Conclusion:** Our ML has shown high performance in AD diagnosis and prediction of MCI progression. It outperformed expert radiologists, and is expected to provide support in clinical practice.

## Introduction

Approximately 50 million people have dementia worldwide, with almost 10 million new cases annually. Alzheimer's disease (AD) is the most common form of dementia and contributes to well over 50% of cases. In machine learning (ML), data are transferred by functions into a high-dimensional mathematical space to properly form clusters. The number of dimensions corresponds to the number of concerned variables, and ML enables the handling of a large number of variables to analyze the data. In high-dimensional space, it is easier to find a separable boundary. However, as the dimension increases, the data become sparse, and more data are needed to obtain an optimal solution. This is known as the “curse of dimensionality.” In situations where the amount of available data is limited, it is necessary to devise an appropriate way to incorporate the configuration of concerned variables to reduce the number of variables used. If the data scientist is familiar with the meaning of the dataset and its numerical features, important variables can be selected intentionally. An alternative method is using prior operations, such as voxel-based morphometry (VBM), which normalizes the brain shape and scales the regional brain volumes by adjusting for brain size, age, gender, etc. Selecting which ML algorithm to use is crucial, as each has its own strengths and weaknesses. For example, when analyzing a large amount of data, deep learning (DL) often outperforms other alternative ML algorithms through representation learning and self-optimization. However, the learning process of DL cannot be fully interpreted; it is difficult to validate the results, and the preparation of a large amount of data is a practical challenge. When the data size is small (~1,000), a support vector machine (SVM) can perform well when combined with prior operations such as VBM. As is often the case with medical image data, if the available data are small, it is effective to standardize the data by VBM prior to machine learning. Here, we introduce a software named brain anatomical analysis using diffeomorphic deformation (BAAD) that enables image analysis with ML, combining VBM and SVM for the diagnosis of Alzheimer's disease (AD) and prediction of mild cognitive impairment (MCI) progression to AD.

**Figure 1:**
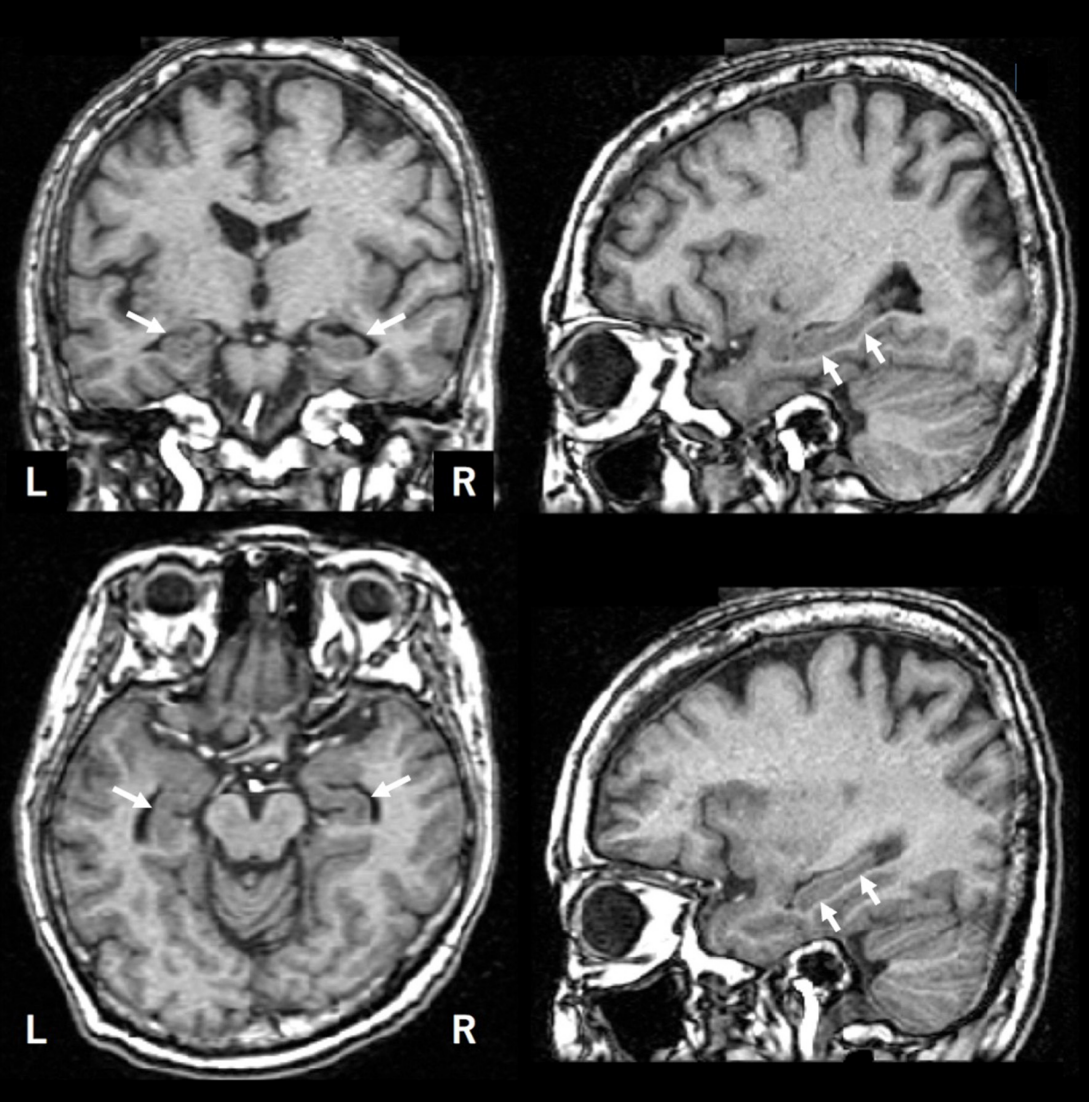
An illustrative brain MRI of a patient with complainant of memory impairment. A 56-year-old woman. After her retirement, she noticed her forgetfulness. She soon forgot what she had done and began to ask the same questions over and over. Later, as her symptoms gradually progressed, she was escorted to the hospital by her family. Her MMSE score was 28 points. The hippocampus is indicated by arrows.

Hippocampal atrophy is one of the diagnostic biomarkers for AD; however, how do clinicians objectively evaluate it (see Figure 1). There are several ways to measure the hippocampal volume including manual delineation, automated techniques, and qualitative ratings. Several reports have proven that manual and automated measurements are well-correlated to one another (1, 2). In this study, we used a software named voxel-based specific regional analysis system for Alzheimer's disease (VSRAD) as an example of an automated technique, which has been widely used in clinical practice in Japan. The details of the method are available elsewhere (3). VSRAD has a region of interest (ROI) in the medial temporal structures, such as the entorhinal cortex, hippocampus, and amygdala, where atrophy is common in AD patients. VSRAD presented a z-score of the ROI by comparing individuals to healthy subjects aged 54–86 years, with a mean age of 70.4 ± 7.8 years (4). The illustrative case in Figure 1 is of a 56-year-old woman who began to complain of subjective memory disturbance after retirement. VSRAD showed z-scores of 1.25 and 1.47 for the left and right medial temporal regions, respectively, suggesting mild shrinkage of the regions.

The BAAD software developed in this study (http://www.shiga-med.ac.jp/~hqbioph/BAAD(English)/BAAD.html) also uses VBM but includes algorithmic analysis not found in VSRAD. In the case shown in Figure 1, BAAD shows z-scores of 2.78 and 3.70 in the left and right hippocampi, respectively. The reason for the difference in *z*-scores between VSRAD and BAAD was partly owing to the differences in VBM procedures and ROI location, but mainly because of the statistical design. The mean age of the reference group provided by VSRAD was approximately 70 years, which is relatively higher than the subject's age of 58 years, resulting in underestimation of brain atrophy of the ROIs. BAAD uses age as a covariate, resulting in age corrected z-scores of the hippocampi that are more accurate for the subject's age. As shown in this example, when assessing hippocampal atrophy, we may unknowingly compare the size with that of a subject of approximately 70 years old, which is common in AD patients. Moreover, the ML in BAAD showed an AD likelihood score of 0.944 (max = 1), indicating high probability of AD. This ML predicts AD based on information in entire brain regions as well as the hippocampus.

## Materials and Methods

### Procedures of VBM and Regional Brain Volume Evaluation in Each Individual of BAAD

**Figure 2:**
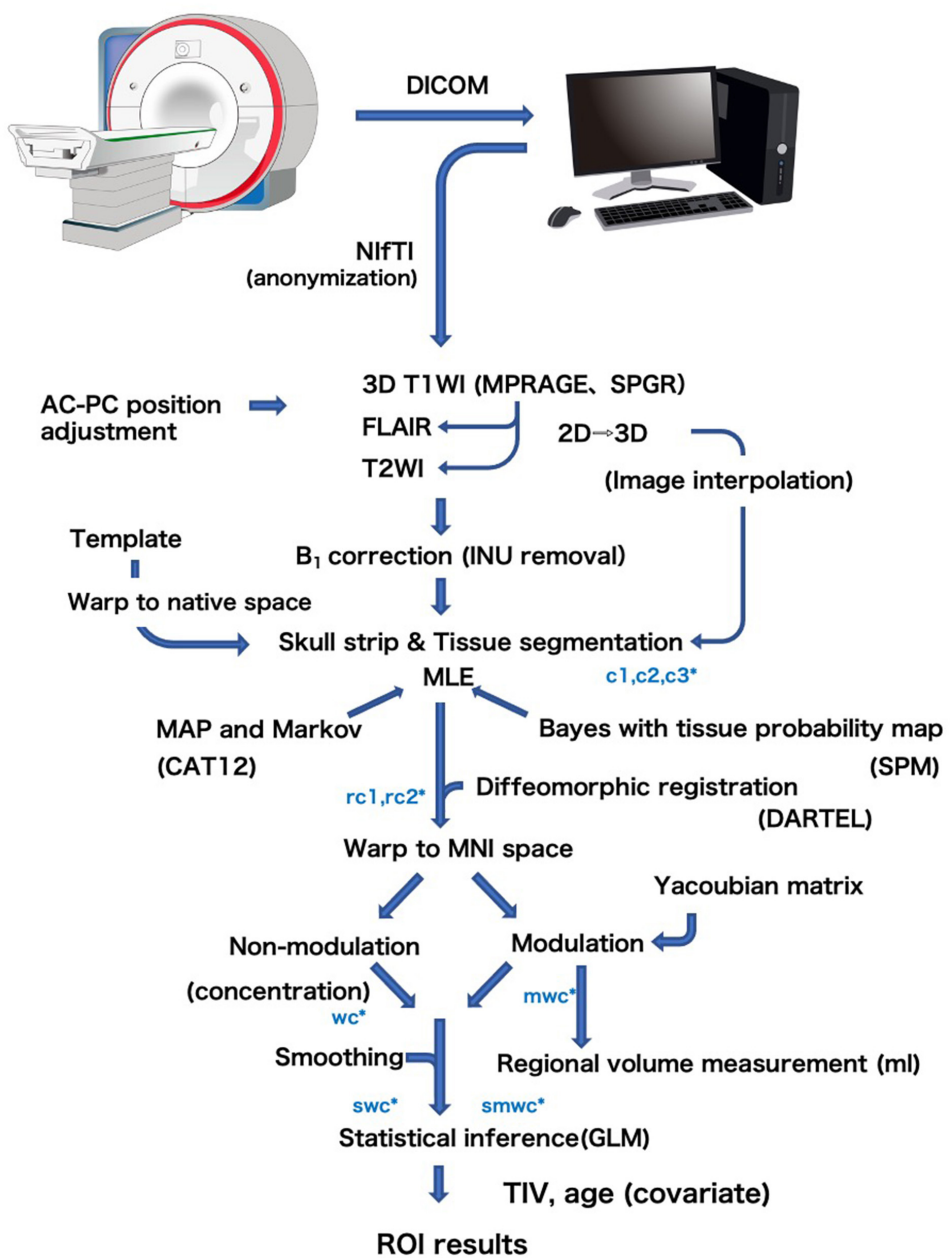
General flow diagram of VBM procedure of the BAAD. Brain is extracted from 3D MR images by skull striping, segmented into gray matter (c1), white matter (c2), and cerebrospinal fluid (c3), and warped into MNI space (w) using DARTEL algorithm. The change of a voxel volume is converted into a voxel signal by “modulation” procedure (m). Finally the data are smoothed to obtain smwc (smoothed, modulated, warped, segmented) images.

VBM is a computational morphometric analysis to evaluate regional brain volume and statistically examine the shape of the brain, using statistical parametric mapping (SPM) software, as first proposed by Ashburner et al. (5). The flow of VBM processing in the BAAD software is shown in Figure 2. Details of the standard VBM procedure are available elsewhere (6). The brain is extracted from 3D MR images by skull striping, segmented into gray matter (GM), white matter (WM), and cerebrospinal fluid (CSF), and warped into Montreal Neurological Institute (MNI) space. Tissue segmentation and intensity of non-uniformity (INU) removal are performed using Computational Anatomy Toolbox (CAT) 12 developed by the Structural Brain Mapping Group at the University of Jena. This toolbox is designed to be an extension of the segmentation in SPM12, and there are several different ways to segment. The most notable difference is the adoption of an Adaptive Maximum A Posterior (AMAP) technique which avoids using prior probability information for tissue probabilities (7). Prior probabilities are derived from a large number of “normal young” subjects and are used for Bayesian rules to assign the probability that each voxel belongs to a given tissue class. The obtained posterior probabilities are treated as partial volumes in SPM. This processing causes standardization of the segmented images, risking the of trimming outliers that may result from pathological changes of the brain. Therefore, tissue probability maps were only used for spatial normalization, initial skull stripping, and initial segmentation estimation. The distribution of signal intensity in GM may differ between cortical surface and deep structures, such as the basal ganglia and thalamus. Therefore, Gaussian segmentation is performed separately in surface and deep regions. For noise reduction, a spatial-adaptive Non-Local Means (SANLM) denoising filter (8) and Markov Random Field (MRF) approach are applied during AMAP segmentation.

Coordinate transformation from native space to MNI space was performed using Diffeomorphic Anatomical Registration Through Exponentiated Lie Algebra (DARTEL) algorithm (9). This is a type of large deformation diffeomorphic metric mapping (LDDMM) that allows processing of brains with large deformations, such as atrophic brains. During the coordinate transformation, it is necessary to reflect the volume change of the voxels according to the migration distance of each voxel caused by the brain deformation. In this transformation procedure, the change in voxel volume is converted into a voxel signal. The templates for DARTEL are created from 550 healthy control subjects of the IXI database.

To reduce the number of feature vectors for the SVM classifier, BAAD uses multiple sets of adaptive ROIs instead of the voxel-wise method (10). There are two ways to obtain the values of the ROI; averaging the values of voxels in the ROI, and the ROI-wise method using ROI as a unit. Theoretically, the results of these methods should be the same, but the voxel-wise method is more susceptible to noise than the ROI-wise method. Therefore, BAAD adopted the MarsBar toolbox (http://marsbar.sourceforge.net) to perform ROI-wise analysis using preset ROIs for automated anatomical labeling (AAL), Brodmann's atlas, and the LONI Probabilistic Brain atlas (LPBA40). Each atlas has ROIs of 108, 118, and 56, respectively. In this process, local volume was adjusted by total intracranial volume (TIV) and age. We did not include sex as a covariate, as it was empirically known that sex differences were offset by TIV. For *z*-score estimation, BAAD used subjects over age 50 in the IXI database as a reference, with age and TIV as confounding factors. *Z*-scores were derived from ([control mean] – [individual value])/(control standard deviation).

### ADLS and Hyperparameter Tuning of SVM

**Figure 3:**
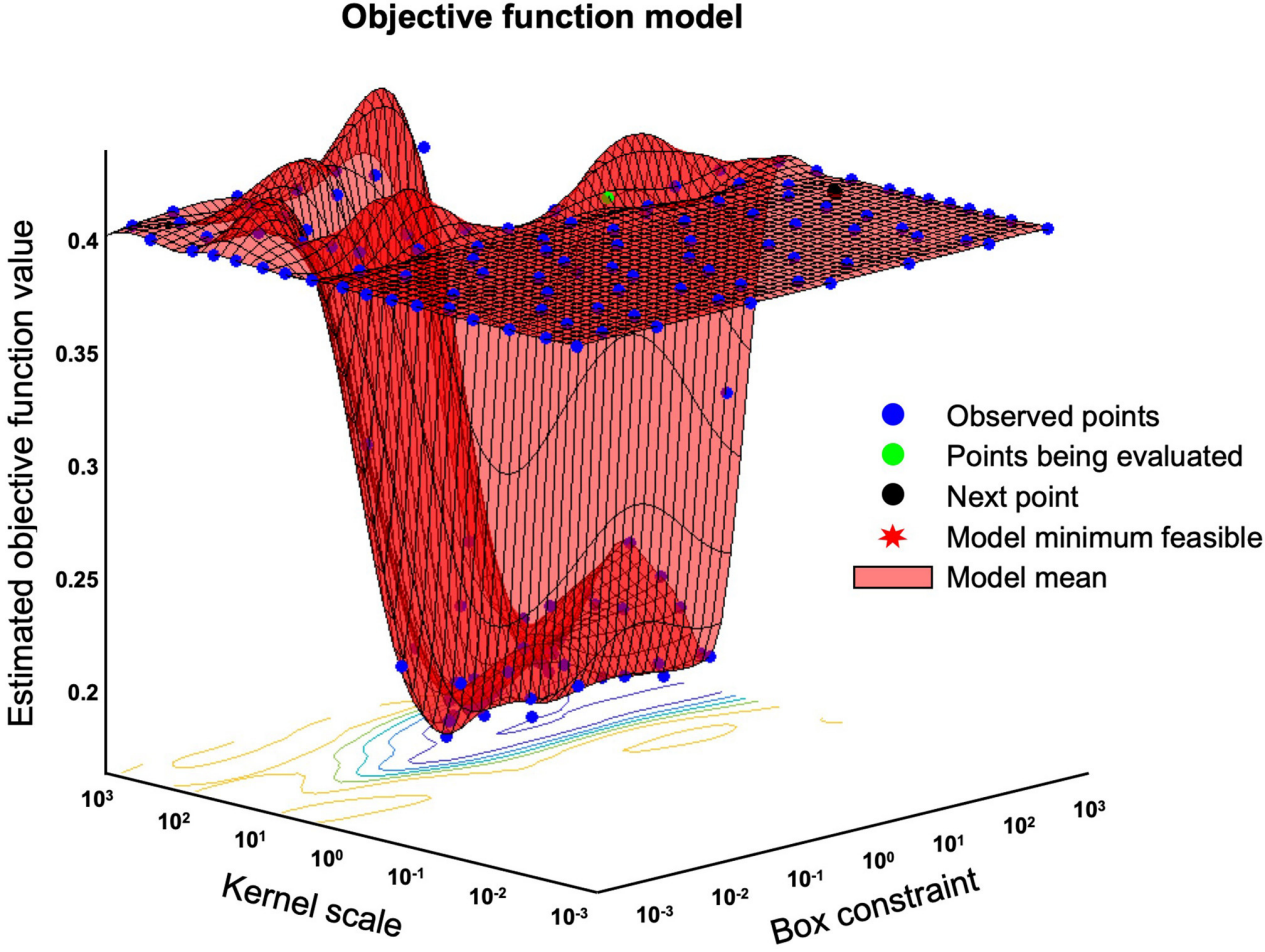
Bayesian optimization of “bayesopt” in MATLAB. This figure shows an example of a Bayesian optimization object by using bayesopt to minimize cross-validation loss. Optimize hyperparameters of a *k* nearest neighbors (KNN) classifier for the ionosphere data, that is, find KNN hyperparameters that minimize the cross-validation loss.

We expressed the likelihood of the AD brain as the Alzheimer's disease likelihood score (ADLS), which represents the distance to the separating hyperplane. ADLS is obtained from the posterior probability function, *Pr* = (*Y* = *k*|*X* = *x*), where the probability *Y* is the class *k* given that the input variable *X* is *x*. The probability is transformed by a sigmoid function so that the value is within the range of [0, 1]. The larger the value, the higher the likelihood of AD. We used radial basis function (RBF) for the SVM kernel, and the values of parameters were optimized using the Alzheimer's disease neuroimaging initiative (ADNI) database. The basic principal of SVM is explained in the Supplementary Section. For training and validation, we introduced a spatial-anatomical method by providing the z-score of the ROIs as a feature vector. Leave-one-out cross validation was used to obtain unbiased estimates and to avoid over-fitting in this procedure. A mathematical procedure was performed using the “fitcsvm” function implemented in MATLAB R2016b (MathWorks, Natick, Massachusetts, USA). The sequential minimal optimization (SMO) algorithm and “bayesopt” were used for the optimization of the parameters (Figure 3). Finally, the created model was used in the validation dataset to fine-tune the model hyperparameters by comparing it to the training dataset. The validation dataset helps to refine the range of hyperparameters in the model and ultimately provides an unbiased fitting model on the dataset.

### Participants and Data Source

We used ADNI data from three countries; North America (NA-ADNI), Australia (Australian Imaging, Biomarker & Lifestyle Flagship Study of Aging AIBL)), and Japan (JADNI). Data from North America and Australia were extracted from the LONI Image and DATA Archive (ida.loni.usc.edu). Data from Japan were extracted from the National Bioscience Database Center (hum0043.v1). More information about the database can be found at adni-info.org. The NA-ADNI was launched in 2003, and started in 2004 as a longitudinal, multicenter cohort study of healthy elderly and individuals with MCI and early AD. AIBL and JADNI were launched in 2006 and 2007, respectively. More information on the diagnostic criteria can be found on the ADNI web site (http://www.adni-info.org/Scientists/AboutADNI.aspx).

We selected 1,446 subjects from NA-ADNI database consisting of 543 cognitively normal (NL), 544 MCI and 359 AD participants. We classified MCI patients into progressive MCI (pMCI) and stable MCI (sMCI), depending on the progression to AD or not. Of the sMCI cases, 132 subjects were excluded because of the short observation period (<4 years), which will be discussed in more detail later in the paper. The training set consisted of 723 randomly selected subjects, and the remaining 723 subjects created the validation set (Table 1). To verify the generalization capability of the ML, we used a database of 519, 592, 69, and 128 adults over 60 years from the AIBL, JADNI, Minimal Interval Resonance Imaging in Alzheimer's Disease (MIRIAD), and Open Access Series of Imaging Studies (OASIS) cross-sectional databases, respectively (Table 2). The MIRIAD study was designed to investigate the feasibility of using magnetic resonance imaging (MRI) as an outcome measure in clinical trials for AD treatment. Details of the study can be found at the MIRIAD web site (https://www.ucl.ac.uk/drc/research/methods/minimal-interval-resonance-imaging-alzheimers-disease-miriad). OASIS provides freely available data and the details are available elsewhere (11).

**Table 1:** Demographic features of the North American ADNI.

**ADNI (North America)**		**AD spectrum**				**Non-AD spectrum**			
		**AD (359)**		**pMCI (284)**		**sMCI (128)**^**+**^		**NL (543)**	
Number of subjects		179	*180*	142	*142*	64	*64*	271	*272*
Age		75.8 ± 7.9	*74.8 ± 7.9*	74.4 ± 6.9	*74.0 ± 7.4*	72.7 ± 7.5	*72.4* ±*8.1*	75.0 ± 5.8	*73.5* ±*5.8*
Sex (M/F)		106/73	*88/92*	89/53	*82/60*	45/19	*35/29*	139/132	*122/150*
MMSE		23.2 ± 2.1	*23.2* ±*2.0*	26.8 ± 1.8	*26.9* ±*1.7*	27.9 ± 1.6	*28.2* ±*1.8*	29.0 ± 1.1	*29.1* ±*1.2*
ApoE^*^ num (%)	22	1	(25.0)	0		0		3	(75.0)
	23	10	(11.0)	6	(6.6)	8	(8.8)	67	(73.6)
	33	103	(18.1)	85	(15.0)	75	(13.2)	305	(53.7)
	24	7	(26.9)	7	(26.9)	5	(19.2)	7	(26.9)
	34	159	(34.4)	139	(30.1)	34	(7.4)	130	(28.1)
	44	66	(50.4)	47	(35.9)	6	(4.6)	12	(9.2)

*ADNI, Alzheimer's disease neuroimaging Initiative; AD, Alzheimer's disease; MCI, mild cognitive impairment; pMCI, progressive MCI; sMCI, stable MCI; NL, normal subjects; MMSE, Mini-Mental State Examination; ApoE, apolipoprotein E*.

+ *, First 260 subjects were selected as sMCI, later 132 subjects were removed because of short observation periods*.

* *, ApoE was not examined in 13 AD and 19 NL*.

*Italic numbers are subjects selected for validation*.

**Table 2:** Demographic features of the subjects used in this study.

	**AIBL**		**JADNI**				**MIRIAD**		**OASIS**	
Group	NL (447)	AD (72)	NL (165)	AD (158)	sMCI^*^(129)	pMCI (140)	NL (23)	AD (46)	NL (98)	AD (30)
Age	72.4 ± 6.2	73.1 ± 7.9	68.2 ± 5.6	74.1 ± 6.6	72.8 ± 6.0	73.6 ± 5.6	69.7 ± 7.2	69.5 ± 7.0	75.9 ± 9.0	78.0 ± 6.9
sex (M/F)	192/254^+^	30/42	78/87	68/90	76/53	59/81	12/11	19/27	26/72	10/20
MMSE	28.7 ± 1.2	20.5 ± 5.6	29.2 ± 1.2	22.5 ± 1.8	26.8 ± 1.9	26.0 ± 1.6	29.5 ± 0.9	17.4 ± 5.8	29.0 ± 1.2	21.2 ± 4.0

*AIBL, Australian Imaging, Biomarker & Lifestyle Flagship Study of Aging; JADNI, Japanese Alzheimer's disease neuroimaging Initiative; MIRIAD, Minimal Interval Resonance Imaging in AD; OASIS, Open Access Series of Imaging Studies; MMSE, Mini-Mental State Examination; AD, Alzheimer's disease; NL, normal subjects; sMCI, stable MCI; pMCI, progressive MCI*.

* *, Not meeting the criteria being stable for 4years or more*.

+ *, Sex of one subject was unknown in AIBL study*.

*The pairs in highlighted box are p < 0.001 (t-test)*.

Structural MR images of the brain were acquired with 1.5T (ADNI1, AIBL, JADNI) and 3.0T (ADNI-GO, ADNI2) scanners from several vendors including Philips Medical Systems, Siemens and GE healthcare. All images were scanned under the ADNI protocol conditions with 3D-sagittal plane slices and magnetization prepared rapid acquisition gradient echo sequence (MPRAGE) sequence (12). In the case of the GE scanner, the MPRAGE was used for the ADNI-1 phase, then switched to fast spoiled gradient echo with an inversion recovery preparation (IR-FSPGR) sequence for the ADNI-GO and ADNI-2 phases. The details of the MRI protocol are available elsewhere (13). In the MIRIAD study, brain structural MR images were acquired using an IR-FSPGR sequence on a 1.5 T Signa MRI scanner (14). In the OASIS study, brain structural MR images were acquired using the MPRAGE sequence on a 1.5 T Siemens Vision scanner.

### Machine Learning for AD Spectrum

MCI is a transitional stage between normal aging and dementia. It is estimated that between 5 and 20% of people over 65 years of age have MCI, and the annual rate of progression to AD is 10–15% among persons with amnestic MCI (15). However, it has become apparent that only 50% convert to AD even in a long-term follow-up period (16). The 3-year progression rates to AD were 50 and 49% in prodromal AD meeting the IWG-1 criteria, and intermediate AD likelihood meeting the NIA-AA criteria, respectively (17). In this study, using the NA-ADNI database, 52.2% (284) of 544 MCI patients progressed to AD. Of the MCI patients who converted to AD during follow-up, 87.3% converted within 1 year, and 95.8% converted within 3 years. Therefore, we defined sMCI as MCI patients who had not progressed for 4 years and more. Of the 260 sMCI subjects initially selected, 132 with <4 years of follow-up were excluded, and of the remaining 128 sMCI subjects, 65 were grouped for training and 63 for evaluation (Table 1). The observation period for pMCI ranged from 6 to 108 months, with a mean (SD) of 43.0 (23.6) months and a median of 36 months. The observation period for sMCI ranged from 48 to 120 months, with a mean (SD) of 63.0 (21.8) months and a median of 60 months.

AD can be conceptualized as a biological and clinical continuum from the preclinical phase (clinically asymptomatic subjects with AD pathology) to the clinical phase. Therefore, the preclinical and clinical phases are a seamless sequence. Diagnosis of MCI can be accomplished according to prescribed criteria at the research level, but it may be difficult to implement the same complex protocols in clinical practice. Therefore, it is better not to dwell on the distinction between MCI from normal (NL) or AD. For this reason, we labeled AD and pMCI as being in the AD spectrum, and NL and sMCI as being in the non-AD spectrum for SVM learning. We randomly extracted 321 AD spectrum (179 AD and 142 pMCI) and 335 non-AD spectrum images from the NA-ADNI database (271 NL and 64 sMCI) (Table 1), and used this dataset to train the SVM, named SVMst. A Mini-Mental State Exam (MMSE) score was added for SVM learning, named SVMcog, because the assessment of cognitive ability was important for the diagnosis of dementia. The rest of 322 AD spectrum and 336 non-AD spectrum images were used as validation set to fine-tune the hyperparameters.

### Neuroradiologists

Two neuroradiologists (RI and HK) independently and blindly reviewed the structural MR images. Both neuroradiologists are board-certified experts in Japan with more than 20 years of clinical experience. MR images of 100 AD and 100 NL subjects were randomly selected from the NA-ADNI database, and any information other than age and gender of the subjects was not disclosed to the radiologists. First, 10 AD and 10 NL subjects were extracted from the sets and used for training. Second, a few days later, the radiologists were asked to diagnose AD or NL from the 200 structural images using MRIcron software (University of Nottingham School of Psychology, Nottingham, UK; www.mricro.com). Third, after completion of the initial diagnosis, the radiologists were allowed to modify their diagnoses with reference to the results of VSRAD.

### Amyloid Positron Emission Tomography (PET)

Amyloid PET images were obtained from different PET scanners, manufactured by GE, Siemens, and Philips. The dynamic 3D acquisition method was used, consisting of 4 frames of a 5-min scan during 30~60 min interval after the intravenous injection of 370 MBq (~10 mCi) of ^18^F-AV-45 (florbetapir). To define four cortical gray matter regions (frontal, anterior/posterior cingulate, lateral parietal, lateral temporal), brain MRI for each subject was segmented and parcellated with FreeSurfer (version 5.3.0). The standardized uptake value ratio (SUVR) was calculated by creating a conventional (non-weighted) average across the four main cortical regions and was normalized by the entire cerebellum reference region. Data were downloaded from the LONI web site (https://ida.loni.usc.edu/login.jsp). The SUVR cutoff value was defined above 1.11 according to the recommendation of UC Berkeley (18).

### Statistical Analyses

Kaplan-Meier and other statistical analyses were performed with JMP® software (version 14.3, SAS Institute, Cary, NC). The receiver operating characteristic (ROC) curve was used to evaluate the ability of the model for classifying disease. To obtain maximum potential effectiveness, optimal cut-off point was determined by Youden Index. The equations of accuracy, sensitivity, specificity, positive predictive value (PPV), negative predictive value (NPV), F1, and Matthews correlation coefficient (MCC) are available elsewhere (19, 20). MCC is the geometric mean of the regression coefficients of the problem and its dual, and is robust against imbalanced classes. A significance level was set at *p* < 0.05.

## Results

### AD-NL Classification by BAAD ML

**Figure 4:**
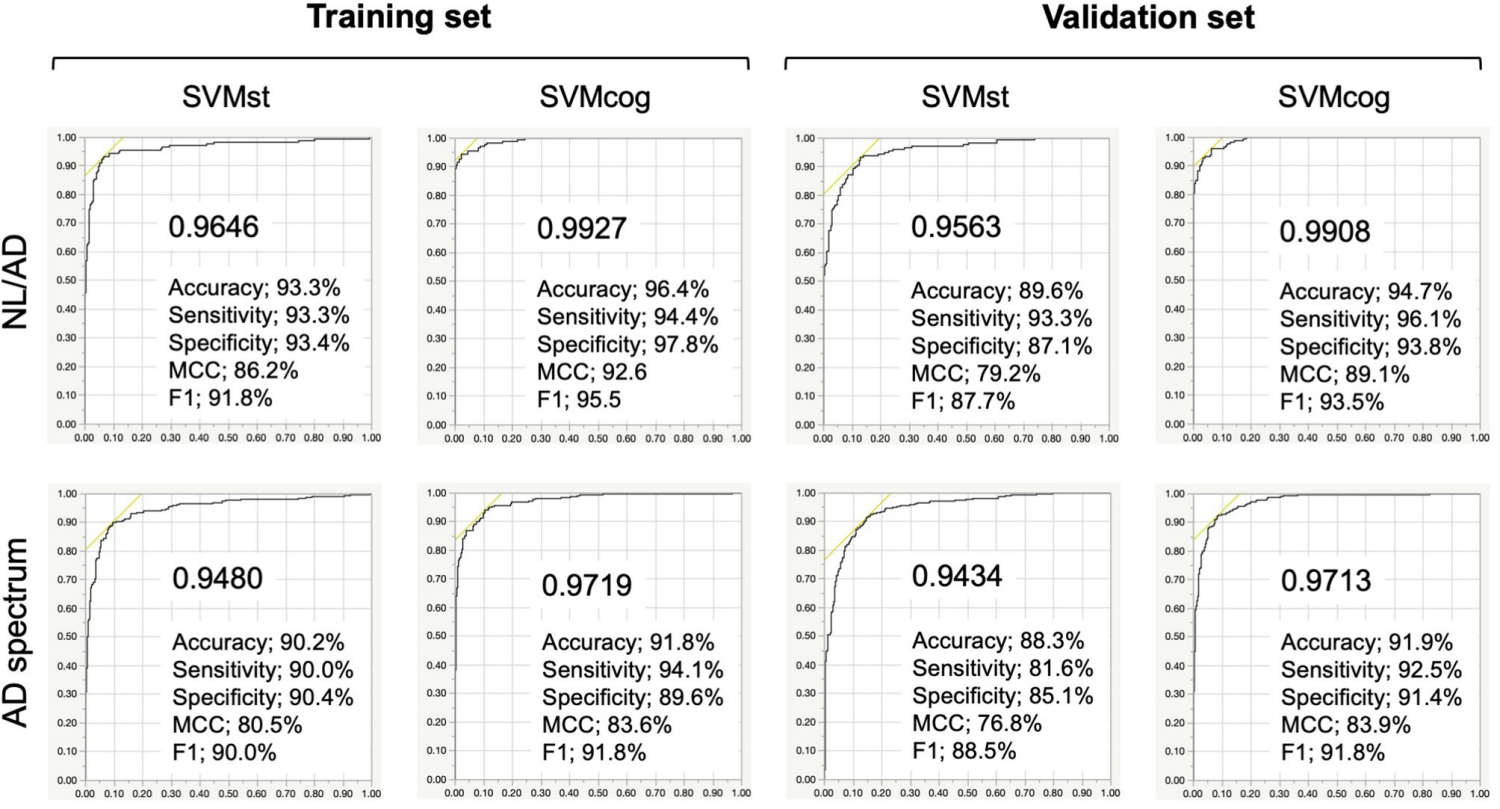
Performance of the SVMs in training and validation set from NA-ADNI. Upper row, results of separation between normal (NL) and Alzheimer's disease (AD) of the SVMst and SVMcog. Lower row, results of separation between AD spectrum and non-AD spectrum. Numerical values in the graph indicate area under the curve (AUC). MCC, Matthews correlation coefficient. The optimal cut-off point was determined by Youden Index.

The results of the ML performance for AD diagnosis in the NA-ADNI (training and validation set, see Table 1) are shown in Figure 4. We showed the ROC curves and the AUC to illustrate the performance of the algorithm. The values for diagnostic accuracy were obtained according to the optimal cutoff value derived from Youden's index. Both SVMst and SVMcog showed very high accuracy for AD/NL or spectrums of AD/non-AD classification. The hyper-parameters of SVMs appear to be adequately optimized because the area under the curve (AUC) value did not change drastically in the validation set.

**Figure 5:**
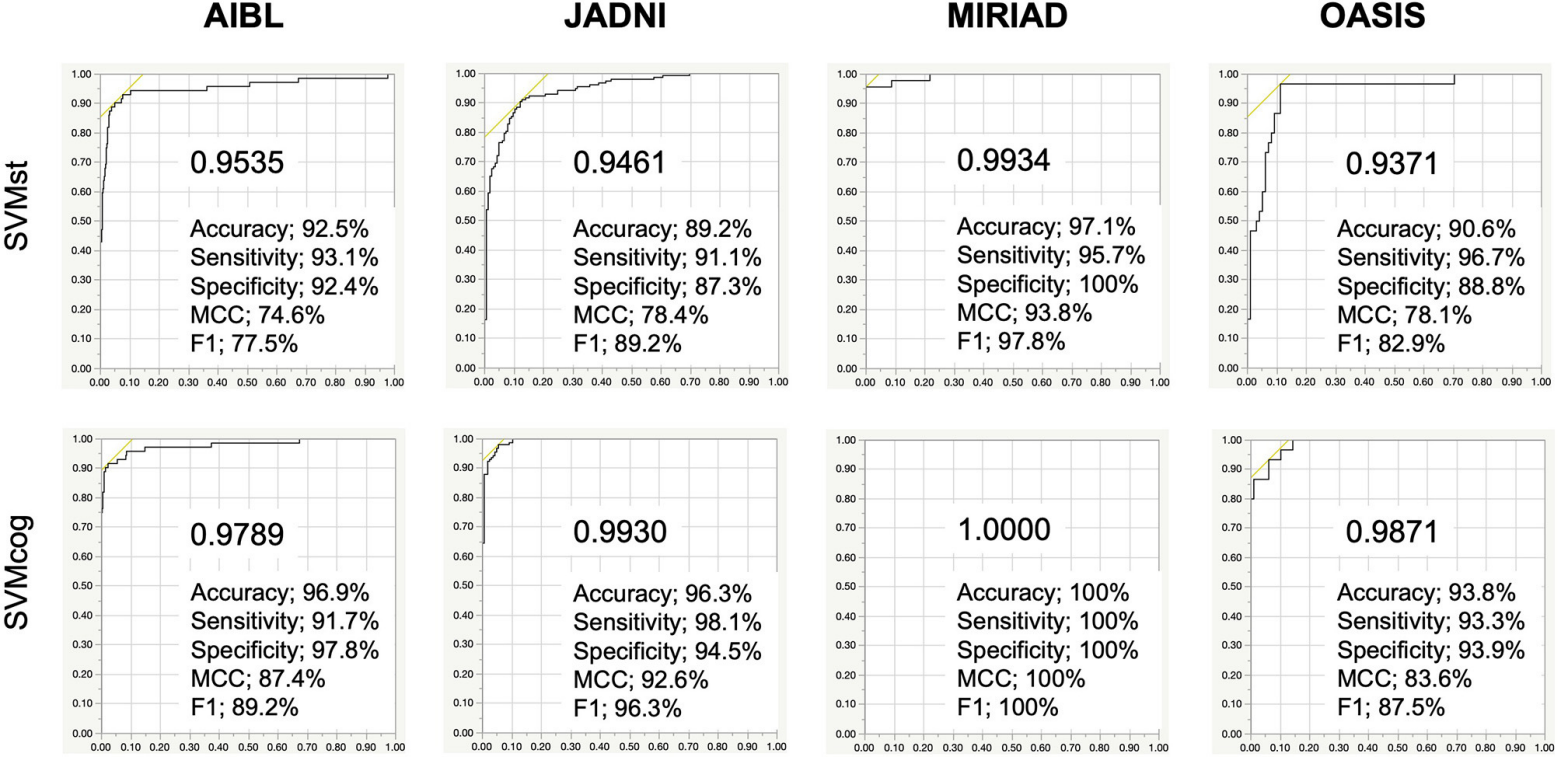
Performance of the SVMs in several cohorts. The cutoff in ADNI, AIBL, JADNI, and MIRIAD uses the respective optimal values. AIBL, Australian Imaging, Biomarker & Lifestyle Flagship Study of Aging; JADNI, Japanese ADNI; MIRIAD, Minimal Interval Resonance Imaging in AD; OASIS, Open Access Series of Imaging Studies. Numerical values in the graph indicate area under the curve (AUC). MCC, Matthews correlation coefficient.

The performance of the SVMs in other databases (test datasets, see Table 2) is shown in Figure 5. The classification accuracy for SVMst was approximately 90%. The diagnostic accuracy of the SVMcog became higher than that of SVMst by using the MMSE score, maintaining 95% in all databases. The performance was recalculated with the cuttoff value of ADLS > 0.5 because the optimal cut-off value for AD diagnosis of ADLS in the NA-ADNI database was 0.48, and the results are summarized in Table 3. The classification accuracy was ranged from 88.0 to 94.2% and 92.5 to 100% for SVMst and SVMcog, respectively.

**Table 3:** Diagnostic ability for AD/NL by SVMst and SVMcog in several cohorts.

	**SVMst (0.5)**					**SVMcog (0.5)**				
**Database**	**NA-ADNI**	**AIBL**	**JADNI**	**MIRIAD**	**OASIS**	**NA-ADNI**	**AIBL**	**JADNI**	**MIRIAD**	**OASIS**
AD/NL	180/272	72/447	158/165	46/23	30/98	180/272	72/447	158/165	46/23	30/98
AUC (%)	0.9563	0.9535	0.9461	0.9934	0.9371	0.9908	0.9789	0.9930	1.0000	0.9871
Accuracy (%)	88.9	89.2	88.0	94.2	90.6	93.8	92.5	95.3	100.0	93.0
Sensitivity (%)	87.2	94.4	85.1	97.8	96.7	93.3	93.1	95.3	100.0	93.3
Specificity (%)	90.1	88.4	90.8	87.0	88.8	94.1	92.4	95.4	100.0	92.9
PPV (%)	85.3	56.7	90.0	93.8	72.5	91.3	66.3	95.3	100.0	80.0
NPV (%)	91.4	99.0	86.3	95.2	98.9	95.5	98.8	95.4	100.0	97.8
F1 (%)	86.3	70.8	87.5	95.7	82.9	92.3	77.5	95.3	100.0	86.2
MCC (%)	77.0	67.9	76.1	86.9	78.1	87.1	74.6	90.7	100.0	81.9

*The numbers in parentheses indicate the cutoff value*.

*AD, Alzheimer's disease; NL, Normal subjects; ADNI, Alzheimer's disease neuroimaging Initiative; NA-ADNI, North American ADNI; AIBL, Australian Imaging, Biomarker & Lifestyle Flagship Study of Aging; JADNI, Japanese ADNI; MIRIAD, Minimal Interval Resonance Imaging in AD; OASIS, Open Access Series of Imaging Studies. PPV, positive predictive value (=precision); NPV, negative predictive rate; AUC, area under the curve; MCC, Matthews correlation coefficient*.

The diagnostic accuracy of the two radiologists who reviewed 200 subjects selected from the NA-ADNI database was 57.5 and 70.0%, which was lower than the SVMst at 90.5% (Table 4). After disclosing the results of *z*-value of VSRAD, the accuracy of the radiologists improved to 70.0 and 73.0%, still far short of the SVMst accuracy (90.5%). The kappa coefficient, an index of the degree of concordance between the two radiologists was as low as 0.35 (*p* = 0.710, McNemar test) without VSRAD, but it improved to 0.56 after referring to VSRAD (*p* = 0.016, McNemar test).

**Table 4:** Accuracy of AD diagnosis by two neuroradiologists with and without VSRAD support.

	**Radiologist 1**		**Radiologist 2**		**SVMst**
**VSRAD assistance**	**before**	**after**	**before**	**after**	**–**
Accuracy (%)	57.5	70.0	70.0	73.0	90.5
Sensitivity (%)	55.0	72.0	69.0	67.0	91.0
Specificity (%)	60.0	68.0	71.0	79.0	90.0
PPV (%)	57.9	69.2	70.4	76.1	90.1
NPV (%)	57.1	70.8	69.6	70.5	90.9
F1 (%)	56.4	70.6	69.7	71.3	90.5
MCC (%)	15.0	40.0	40.0	46.3	81.0

*The cutoff value of the SVMst was ADLS > 0.5*.

*PPV, positive predictive value (=precision); NPV, negative predictive rate; AUC, area under the curve; MCC, Matthews correlation coefficient*.

### Prediction of MCI Progression

To assess the ability of the ML to predict MCI progression, we analyzed data from 142 pMCI and 64 sMCI subjects from the NA-ADNI validation set, and the results are summarized in Table 5. The cutoff values were set ADLS > 0.5 for SVMs. The prediction accuracy was 85.4% in SVMst and 87.9% in SVMcog. We also investigated the SVM performance in JADNI. Unfortunately, none of the sMCI met our criteria due to the short follow-up period, therefore, we substituted NL for sMCI. The accuracy of SVMst and SVMcog for classification between normal and pMCI in JADNI were 86.4 and 91.2%, respectively. Since these results were comparable with the accuracy of the results obtained as a reference from NA-ADNI, it was speculated that these SVMs were also useful in JADNI to predict MCI progression.

**Table 5:** Prediction of MCI conversion.

	**NA-ADNI**			NA-ADNI^*^		JADNI^*^	
	**SVMst**	**SVMcog**	**VSRAD**	**SVMst**	**SVMcog**	**SVMst**	**SVMcog**
AUC	0.9064	0.9019	0.7407	0.9400	0.9632	0.9240	0.9697
Accuracy (%)	83.0	85.0	62.1	87.2	90.6	86.4	91.2
Sensitivity (%)	81.7	83.8	54.2	81.7	83.8	81.0	86.0
Specificity (%)	85.9	87.5	79.7	90.1	94.1	90.8	95.4
PPV	92.8	93.7	85.6	81.1	88.1	87.5	93.7
NPV	67.9	70.9	44.0	90.4	91.8	85.7	89.5
F1	86.9	88.5	66.4	81.4	85.9	84.1	89.7
MCC	64.1	67.9	31.6	71.6	78.9	72.5	82.3

*The subjects of the NA-ADNI were selected as validation set*.

*The cut-off values of SVMst and SVMcog were set as ADLS > 0.5*.

* *, discrimination between pMCI and cognitively normal subject*.

*PPV, positive predictive value (=precision); NPV, negative predictive rate; AUC, area under the curve; MCC, Matthews correlation coefficient*.

We assessed the predictive accuracy of the MCI progression by SVMst in the NA-ADNI database for each timing of MRI examinations before the onset of AD and compared it to the predictive accuracy of AV-45 PET (Table 6). As expected, the accuracy of SVMst decreased as the time to onset increased, but the decrease appeared to be small. In this model, the number of cases of sMCI was fixed and the number of cases of pMCI decreased with time to onset. This created a class imbalance, and accuracy was no longer a reliable measure. Therefore, we focused on the values of MCC. The MCC in SVMst worsened with duration but was better than the AV-45 PET at all times up to the onset. The MCC values in SVMst showed a relatively large decrease between 2 and 3 years prior to the onset. The predictive ability of amyloid PET was not expected to be significantly affected by time because Aβ deposition occurs earlier than brain atrophy. However, contrary to our expectations, the MCC in AV-45 decreased with increasing time to onset, with relatively large decreases between 3 and 4 years prior to the onset.

**Table 6:** Prediction for disease progression at each examination timing before onset.

**Time to**	**SVMst (cutoff** **=** **0.5)**				**AV-45 (cutoff** **=** **1.11)**				
**Conversion**	**1 year**	**2 year**	**3 year**	**4 year**	**1 year**	**2 year**	**3 year**	**4 year**	**overall**
pMCI/sMCI	106/128	94/128	48/128	24/128	34/63	28/63	18/63	5/63	85/63
AUC	0.9208	0.9135	0.8667	0.9020	0.8340	0.8220	0.8474	0.7619	0.8287
Accuracy (%)	86.3	83.8	81.3	84.9	71.1	70.3	67.9	61.8	78.4
Sensitivity (%)	85.8	79.8	66.7	75.0	91.2	92.9	94.4	100.0	91.8
Specificity (%)	86.7	86.7	86.7	86.7	60.3	60.3	60.3	58.7	60.3
PPV	84.3	81.5	65.3	51.4	55.4	51.0	40.5	16.1	75.7
NPV	88.1	85.4	87.4	94.9	92.7	95.0	97.4	100.0	84.4
F1 (%)	85.0	80.6	66.0	61.0	68.9	65.8	56.7	27.8	83.0
MCC (%)	72.5	66.7	53.0	53.5	49.7	49.4	45.6	30.8	56.0

*The numbers in parentheses indicate the cutoff value*.

*PPV, positive predictive value (= precision); NPV, negative predictive rate; AUC, area under the curve; MCC, Matthews correlation coefficient*.

### AD Likelihood Score and Brain Aβ Deposition

**Figure 6:**
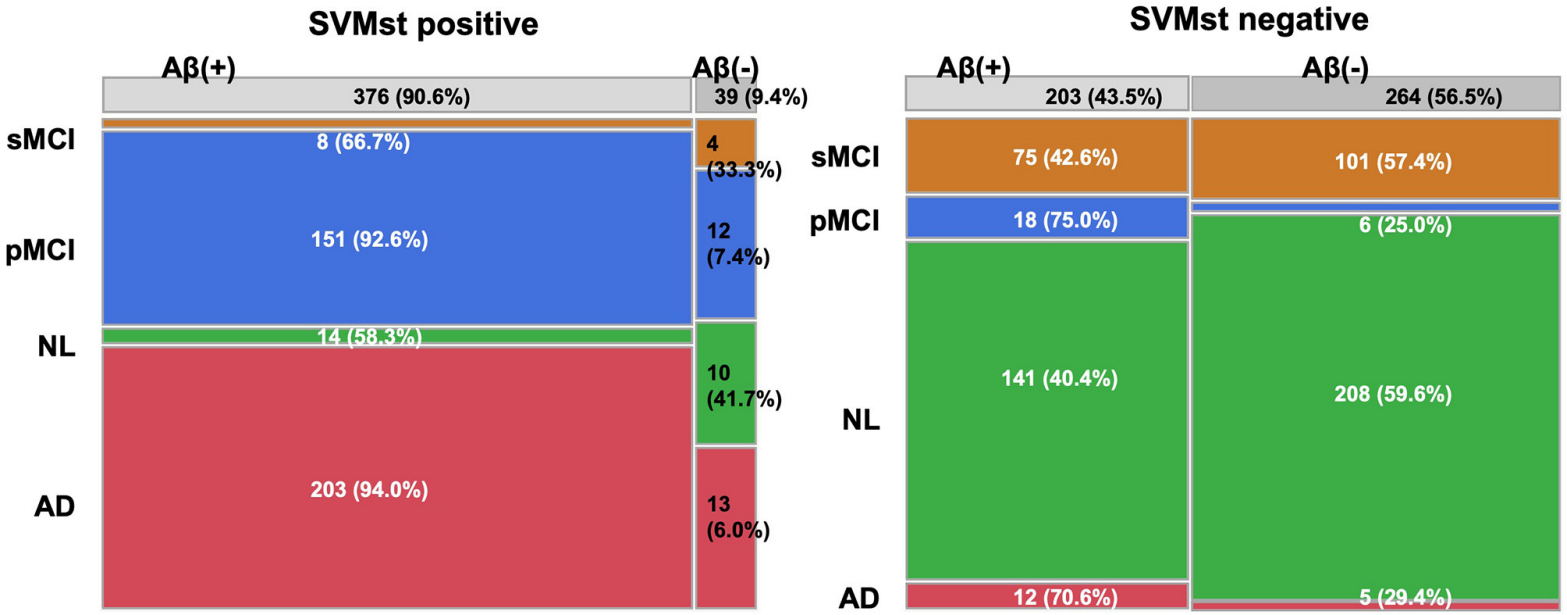
Constitution of diagnosis and amyloid beta deposition in groups classified by SVMst. This figure indicates that SVMst can predict impending dementia with Aβ deposition. AD, Alzheimer's disease; MCI, mild cognitive impairment; pMCI, progressive MCI; sMCI, stable MCI (at least more than 4 years); NL, normal subjects.

The relationship between ADLS and Aβ deposition was investigated in 771 subjects from an AV-45 PET study because Aβ is one of the important molecular targets for disease modifying therapies (DMT). When classified as positive (ADLS > 0.5) by SVMst, 87.1% of the subjects were Aβ positive (SUVR > 1.11) (Figure 6). In SVMst-positive cases, the percentage of pMCI (*n* = 103) of the Aβ-positive MCIs (*n* = 113) was 91.2%, or 92.8% of all pMCI (*n* = 111). In addition, 8 of 11 (72.7%) SVMst-positive MCI patients progressed to AD, even though they were AV-45 negative. For SVMst-negative, 47.1% of the subjects were Aβ positive, and pMCI (*n* = 15) accounted for 23.8% of the total MCI (*n* = 63). When restricted to Aβ-negative MCIs (*n* = 57), the percentage of pMCI (*n* = 6) was 10.5%. These results suggest that there is a strong association between Aβ accumulation and brain atrophy in AD-like patterns. Note that 11.7% patients clinically diagnosed with AD were Aβ negative. These patients may later become Aβ positive, or if not, this indicates the difficulty of AD clinical diagnosis.

## Discussion

This study showed that the proposed BAAD software provides a useful opportunity to address the diagnosis of AD and prediction of the disease progression in MCI patients. A strict classification between MCI and AD is not always necessary in daily medical care; therefore, the SVM was trained by grouping pMCI and AD as the AD spectrum. Subjects assigned to the AD spectrum by the ML are at high risk of having AD pathology, even if their cognitive decline is not significant. Furthermore, by using the MMSE score, SVMcog classified AD and NL at accuracies ranging from 93.8 to 100% in the test databases. In the SVMst-positive cases, 111 of 124 MCIs (89.5%) progressed to AD, 113 (91.1%) were Aβ positive on AV-45 PET, and 103 (91.2%) progressed to AD. Interestingly, for MCI patients with negative AV-45, 72.7% progressed to AD if the SVMst classified them as being in the AD spectrum. It is debatable whether any patient who has AD pathology is suitable for DMT. However, it is helpful to predict patients at high risk of AD progression to facilitate more disease-specific examinations such as amyloid or tau PET, depending on the target of DMT.

It requires some consideration to apply the proposed model to clinical use because the subjects in the ADNI database have already been selected and simplified by excluding other types of dementia. For example, we need to be aware that the ML is not designed to distinguish AD from other types of dementia. In clinical practice, AD is not diagnosed by MRI alone, rather it is used to exclude brain infarction, tumors, infection, hydrocephalus, and other diseases. If there is any doubt, regional cerebral blood flow (CBF) imaging by single photon emission computed tomography (SPECT) has diagnostic value, particularly in differentiating AD from frontotemporal dementia (21, 22). Dopamine transporter (DAT) imaging is a useful tool to diagnose parkinsonism and its related disorders if dementia with Lewy bodies (DLB) or a mixture pathology of DLB is suspected. Metaiodobenzylguanidine (MIBG) myocardial scintigraphy is a widely accepted tool for differentiating Parkinson's disease from other Parkinson-related disorders. In addition to neuroimaging, physical and neuropsychological examinations are also important to check a subject's cognitive and physical mobility level. If a subject is suspected to be cognitively impaired, further examination will be performed to identify which cognition domain is impaired.

The BAAD ML outperformed radiologists in the diagnostic accuracy of AD for reviewing structural MRI. VSRAD reduced inconsistency between the two radiologists and improved the diagnostic accuracy. However, the mean diagnostic accuracy of the radiologists was 71.5% even with the support of VSRAD, whereas that of SVMst was 90.5%. The reason for this is that VSRAD relies only on the volume of the medial temporal structures, whereas the SVMst integrates the information from many ROIs covering the entire brain. Age correction for VBM statistics is also a strength of BAAD, as shown for an illustrative case in Figure 1. The use of ML is beneficial in clinical practice because it is impractical to conduct detailed neuropsychiatric tests on all patients, as they take a long time and may cause suffering. For patients who are apparently demented based on their history of illness, a simple cognitive test such as an MMSE would be sufficient and a detailed cognitive assessment could be omitted. In addition to the brain structure, SVMcog supports AD diagnosis using an MMSE score to reach maximum diagnostic accuracy. VBM may reduce the need for glucose PET or CBF SPECT because the pattern of tau uptake on PET imaging appears to be well-matched with regional patterns of atrophy and hypometabolism, or hypoperfusion, across typical and atypical AD patients (23).

In our model, all MR images were preprocessed by VBM to standardize and simplify the features. Specifically, this included ensuring a unified brain shape, converting the volume into signal intensity, and adjusting the brain volume according to TIV and age. Furthermore, the number of features was reduced by using ROIs instead of individual voxels. Unlike other organs, the brain forms a neuronal network and functional localization, and tau pathology of AD are believed to propagate along the neural network (24). We adopted structural parcellations such as the AAL and Brodmann area, which are commonly used in functional MRI studies. These atlases were expected to reflect the unit of the neuronal network to some extent. Multiple atlases were used because regional redundancy or overlapping among ROIs facilitated optimal weight distribution of features during machine learning, supposedly compensating for the discrepancy between anatomical and functional boundaries. There is no doubt that extracting and integrating the information from many parcellated regions at once by surveying the entire brain is beyond human ability.

A recent study of longitudinal tau PET showed that Aβ accumulation promotes tau pathology, and the tau accumulation rate was increased in the entorhinal, fusiform, inferior and middle temporal, temporal pole, retrosplenial, and the posterior cingulate cortices in the early stage of the disease. In cognitively impaired individuals, tau accumulation was found in all regions, but higher rates were in the inferior temporal, superior orbitofrontal, basal frontal (olfactory and gyrus rectus), and middle occipital cortices (25). The study also found that pathological tau accumulation continues in previously involved regions, that is, tau accumulation is uniform throughout the brain and tau does not accumulate in one region at a time or in a start-stop fashion. The rate of tau accumulation is associated with cerebral atrophy (26), so morphological analysis of the entire brain, such as VBM, allows inference of the distribution of tau lesions. In this sense, it can be said that the VBM has a certain “T” element of the ATN system (27) by examining the pathology of tau indirectly, though not specifically molecularly. It should be considered that there are other factors besides tau pathology, such as TDP-43 and ischemia, that contribute to cerebral atrophy, and that not all development of tau lesions is associated with Aβ, but this is beyond the scope of this study.

There are several limitations to this study. First, as is often pointed out in studies using the ADNI database, patients with non-AD dementia are carefully excluded in advance. Therefore, the clinical validity of the AD diagnosis by the model in this study is limited when patients are screened out for other forms of dementia. Second, the definition of sMCI in our study depends on the follow-up period of the study. For example, some patients with MCI may eventually progress to AD if they have a longer follow-up period. Therefore, it is not recommended to use this ML for long-term preventive treatment. It is not possible to accommodate the non-amnestic type because the MCI patients are weighted to the amnestic type in the ADNI study. Third, once the brain structure of the subject is shared by both the training and test datasets, it becomes difficult to articulate model overfitting. We confirmed the performance of the ML on several untrained databases, but this was limited to the case of AD diagnostics. The accuracy of the pMCI prediction in JADNI was the result of replacing sMCI with NL; therefore, a future goal is to accumulate observed data on sMCI over a long period.

## Conclusion

For a patient with memory disturbance or suspected dementia, it is expected that the proposed ML may support clinicians to diagnose or predict the progression to AD, while the possibility of dementia other than AD should always be kept in mind. For patients with clinically suspected AD, the SVMcog supports AD diagnosis with 95% accuracy, based on MRI and MMSE. Approximately 90% of MCI patients were Aβ-positive when classified as AD spectrum by SVMst. Therefore, these patients should be closely monitored for 3 years or more. Molecular PET examinations will be considered for DMT applications in the future. Currently, there are no codified indicators to accurately predict pathological progression in the prodromal stages owing to the contradictory results regarding the concentration of CSF biomarker proteins (28). Based on the results of this study, our model may facilitate molecular PET for memory loss and dementia patients.

## Data Availability Statement

The raw data supporting the conclusions of this article will be made available by the authors, without undue reservation.

## Ethics Statement

Ethical review and approval was not required for the study on human participants in accordance with the local legislation and institutional requirements. The patients/participants provided their written informed consent to participate in this study.

## Author Contributions

AS was responsible for the project administration, supervision, writing–review and editing. AHS contributed to the data collection, management, and analyses. HK and RI participated in the AD diagnosis of MR images. MI and KT contributed to the research design. All authors contributed to the article and approved the submitted version.

## Conflict of Interest

The authors declare that the research was conducted in the absence of any commercial or financial relationships that could be construed as a potential conflict of interest.

## Supplementary Material

Supplementary Figure 1 Differences in separation boundaries due to dimensional transformation. In a low dimensional (Euclidean) space, it is difficult to create a smooth boundary for complete separation. However, by increasing the dimension, a simple boundary allows for complete separation.
