# Supplementary figures and images for: Machine Learning for Diagnosis of AD and Prediction of MCI Progression From Brain MRI Using Brain Anatomical Analysis Using Diffeomorphic Deformation
Source: Front Neurol. 2021 Feb 5;11:576029. doi: 10.3389/fneur.2020.576029 (PMC7893082; doi:10.3389/fneur.2020.576029)

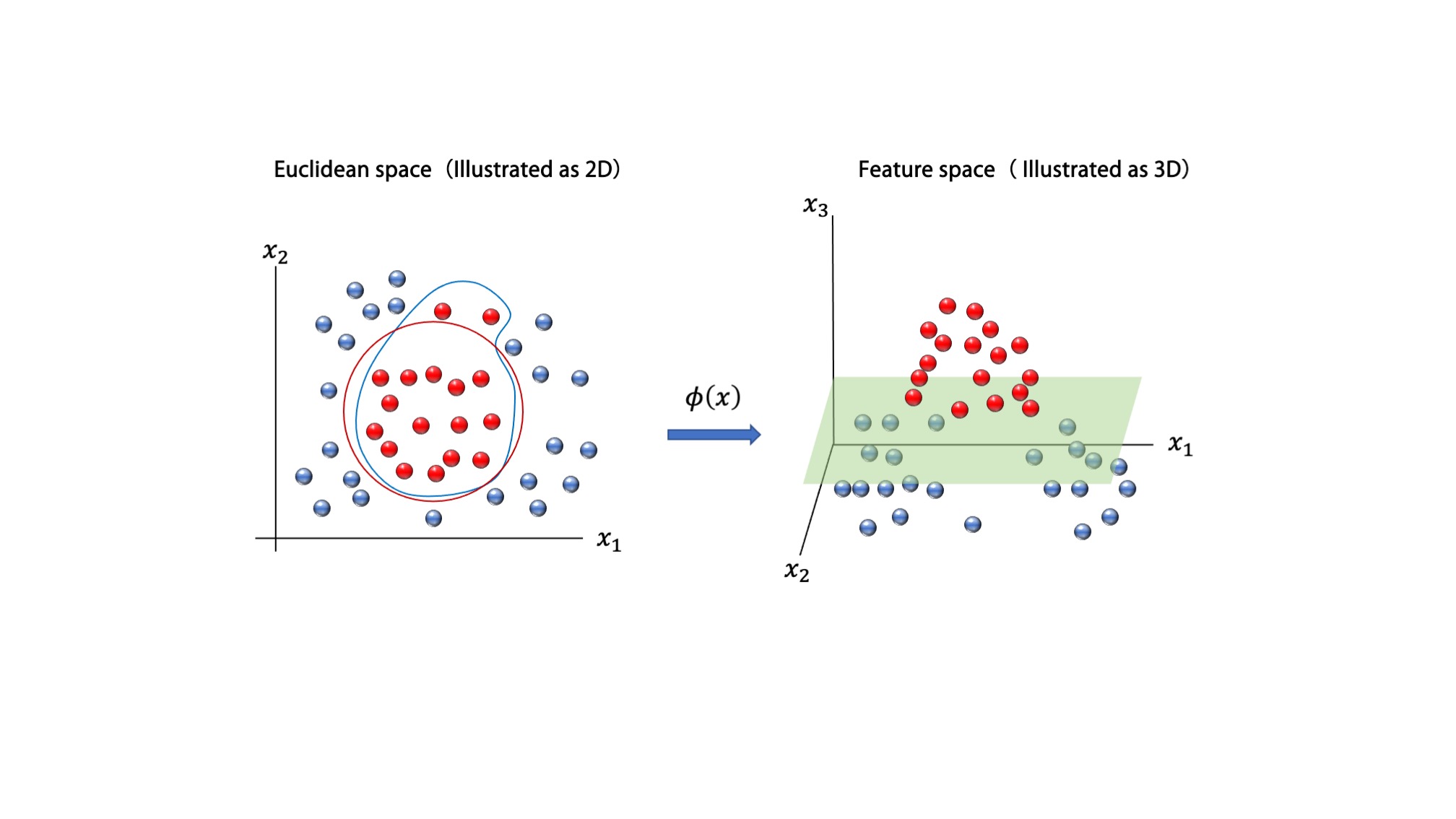

Supplement: Supplementary Figure 1 — Differences in separation boundaries due to dimensional transformation. In a low dimensional (Euclidean) space, it is difficult to create a smooth boundary for complete separation. However, by increasing the dimension, a simple boundary allows for complete separation. [file Image_1.jpeg]
